# Fistule appendiculo-cutanée: complication rare de l'appendicite aiguë

**DOI:** 10.11604/pamj.2014.19.194.4087

**Published:** 2014-10-23

**Authors:** Khalid El Haoudi, Karim Ibn Majdoub

**Affiliations:** 1 Service de Chirurgie Viscérale, CHU Hassan II, Fès, Maroc

**Keywords:** Fistule appendiculo-cutanée, perforation appendiculaire, appendicite aigue, Appendiculo subcutaneous fistula, appendiceal perforation, acute appendicitis

## Image en medicine

La fistule appendiculo-cutanée est une communication anormale entre le tube digestif et la peau à travers l'appendice. Elle représente une complication rare de la perforation appendiculaire au cours de l'appendicite et une urgence chirurgicale. Nous rapportons le cas d'un patient âgé de 48 ans, admis aux urgences pour des douleurs fébriles de la fosse iliaque droite, avec issu de pus à travers un orifice situé au niveau du flanc droit. L'examen clinique avait mis en évidence un orifice de 0,5 cm de diamètre situé au niveau du flanc droit donnant issu à de pus verdâtre, avec une défense de la fosse iliaque, chez un patient fébril à 38,2C. Le bilan biologique avait montré une hyperleucocytose à 15000/mm^3^, avec une CRP à 65 mg/l. Une échographie abdominale a été en faveur d'une appendicite aigue avec un abcès pariétal en regard. Le scanner abdomino-pelvien avait révélé un aspect d'appendicite aigue compliquée d'une fistule appendiculo-cutanée et d'un abcès pariétal en regard (A). Le patient a été opéré par laparotomie médiane avec découverte d'une appendicite aiguë suppurée avec un appendice de 7 cm de long, fistulisé à la peau au niveau du flanc droit, siège d'un abcès pariétal d'environ 20 cc de pus prélevé (B). Le geste a consisté à une appendicectomie rétrograde après déconnexion de la fistule et un drainage de l'abcès. Les suites post opératoires ont été simples et le patient est sorti de l'hôpital à j+4. L'examen anatomo-pathologique de la pièce opératoire est revenu en faveur d'une appendicite aiguë suppurée sans signes de malignité.

**Figure 1:**
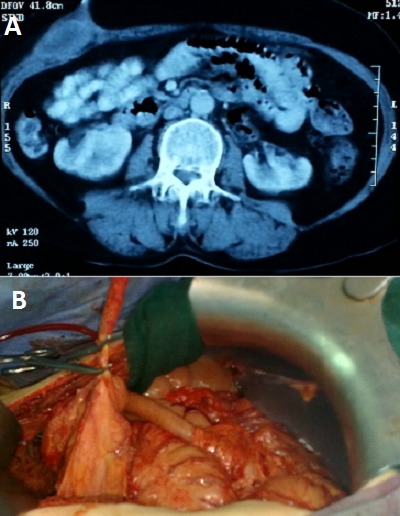
A) aspect scannographique de l'abcès pariétal compliquant la fistule appendiculo-cutanée; B) aspect per opératoire de l'appendice perforé après déconnexion de la fistule appendiculo-cutanée

